# Cumulative incidence of SARS‐CoV‐2 infection in the general population of the Valencian Community (Spain) after the surge of the Omicron BA.1 variant

**DOI:** 10.1002/jmv.28284

**Published:** 2022-11-11

**Authors:** Jorge Camacho, Estela Giménez, Eliseo Albert, Joao Zulaica, Beatriz Álvarez‐Rodríguez, Ignacio Torres, Luciana Rusu, Javier S. Burgos, Salvador Peiró, Hermelinda Vanaclocha, Ramón Limón, María Jesús Alcaraz, José Sánchez‐Payá, Javier Díez‐Domingo, Iñaki Comas, Fernando Gonzáles‐Candelas, Ron Geller, David Navarro, the Valencian Vaccine Research Program (ProVaVac) study group

**Affiliations:** ^1^ Microbiology Service, Clinic University Hospital, INCLIVA Health Research Institute Valencia Spain; ^2^ Institute for Integrative Systems Biology (I2SysBio) University of Valencia‐CSIC Valencia Spain; ^3^ General Directorate of Research and Healthcare Supervision, Department of Health Valencia Government Valencia Spain; ^4^ Foundation for the Promotion of Health and Biomedical Research of the Valencian Community (FISABIO) Valencia Spain; ^5^ General Directorate of Public Health, Department of Health Valencia Government Valencia Spain; ^6^ General Directorate of Healthcare. Department of Health Valencian Government Valencia Spain; ^7^ Preventive Medicine Service, Alicante General and University Hospital Alicante Spain; ^8^ Alicante Institute of Health and Biomedical Research (ISABIAL) Alicante Spain; ^9^ Biomedicine Institute of Valencia, Spanish Research Council (CSIC) Valencia Spain; ^10^ CIBER in Epidemiology and Public Health, Spain; Joint Research Unit “Infection and Public Health” FISABIO‐University of Valencia Valencia Spain; ^11^ Department of Microbiology, School of Medicine University of Valencia Valencia Spain

**Keywords:** cumulative incidence of SARS‐CoV‐2 infection, neutralizing antibodies, SARS‐CoV‐2, seroprevalence, T cells

## Abstract

Studies investigating the cumulative incidence of and immune status against SARS‐CoV‐2 infection provide valuable information for shaping public health decision‐making. A cross‐sectional study on 935 participants, conducted in the Valencian Community (VC), measuring anti‐SARS‐CoV‐2‐receptor binding domain‐RBD‐total antibodies and anti‐Nucleocapsid (N)‐IgGs via electrochemiluminescence assays. Quantitation of neutralizing antibodies (NtAb) against ancestral and Omicron BA.1 and BA.2 variants and enumeration of SARS‐CoV‐2‐S specific‐IFNγ‐producing CD4^+^ and CD8^+^ T cells was performed in 100 and 137 participants, respectively. The weighted cumulative incidence was 51.9% (95% confidence interval [CI]: 48.7–55.1) and was inversely related to age. Anti‐RBD total antibodies were detected in 97% of participants; vaccinated and SARS‐CoV‐2‐experienced (VAC‐ex; *n* = 442) presented higher levels (*p* < 0.001) than vaccinated/naïve (VAC‐n; *n* = 472) and nonvaccinated/experienced (UNVAC‐ex; *n* = 63) subjects. Antibody levels correlated inversely with time elapsed since last vaccine dose in VAC‐n (Rho, −0.52; *p* < 0.001) but not in VAC‐ex (rho −0.02; *p* = 0.57). Heterologous booster shots resulted in increased anti‐RBD antibody levels compared with homologous schedules in VAC‐n, but not in VAC‐ex. NtAbs against Omicron BA.1 were detected in 94%, 75%, and 50% of VAC‐ex, VAC‐n and UNVAC‐ex groups, respectively. For Omicron BA.2, the figures were 97%, 84%, and 40%, respectively. SARS‐CoV‐2‐S‐reactive IFN‐γ T cells were detected in 73%, 75%, and 64% of VAC‐ex, VAC‐n and UNVAC‐ex, respectively. Median frequencies for both T‐cell subsets were comparable across groups. In summary, by April 2022, around half of the VC population had been infected with SARS‐CoV‐2 and, due to extensive vaccination, displayed hybrid immunity.

## 1 INTRODUCTION

Spain has been severely affected by the SARS‐CoV‐2 pandemic, with more than 12.4 million microbiologically confirmed cases as of June 8th, 2022.^1^ The Valencian Community (VC) is the fourth most populous Spanish autonomous community with more than five million inhabitants and has reported around 1.5 million SARS‐CoV‐2 infections and almost 10,000 attributed deaths to date.^1^ Importantly, a large percentage of the VC population has received either a primary full vaccination series (93.9%) or a booster vaccination schedule (57.3%).^2^ Registries of confirmed COVID‐19 cases tend to underestimate the cumulative incidence of SARS‐CoV‐2 infection, as previously shown in the ENE‐COVID study, a nationwide, population‐based, seroepidemiological study conducted in Spain during 2020.^3^

Some serosurvey studies in the general population and extending into the Omicron wave have been reported.^4^, ^5^, ^6^ These studies provided valuable information on the extent of transmission in the past and contributed toward understanding the future course of the pandemic and shaping public health decision‐making. Here, as part of the ProVaVac Valencian COVID‐19 vaccine research program, we conducted a population‐based study aimed primarily at estimating the cumulative incidence of SARS‐CoV‐2 infection in the general VC population after the surge and spread of the Omicron BA.1 variant. Given that both SARS‐CoV‐2‐Spike (S)‐binding functional antibodies and S‐reactive T cells elicited following vaccination or natural infection seemingly contribute toward protecting against severe COVID‐19,^7^, ^8^, ^9^ we also assessed the impact of COVID‐19 vaccination, SARS‐CoV‐2 infection and time elapsed between these two events on SARS‐CoV‐2 humoral and cellular immune status. We specifically measured anti‐SARS‐CoV‐2‐S(Spike)‐Receptor Binding domain (RBD) total antibody levels in all participants, and SARS‐CoV‐2‐S Wuhan‐Hu‐1 (G614), Omicron BA.1 and BA.2 neutralizing antibody (NtAb) titers and SARS‐CoV‐2‐S‐reactive IFN‐γ‐producing CD4^+^ and CD8^+^ T‐cell levels in a subsample of randomly selected subjects, representative of the population recruited.

## 2 METHODS

### 2.1 Study design and setting

The current cross‐sectional, region‐wide study was conducted in the primary care zones (PCZ) of the Valencia Health System in April 2022, after the Omicron BA.1 variant had spread extensively within the VC. A sample size of 998 participants was calculated to achieve a precision of ±1% (Supplementary material). For operational reasons, 100 PCZs were randomly selected to simplify sample transport logistics. Each PCZ was given a specific date on which to collect between 8 and 17 samples depending on the size of the population served, stratified by age and sex. An additional sodium heparin 5 cc tube was collected from patients having blood drawn on doctor's orders for any reason and fulfilling the age/sex selection criteria assigned to each center. The informed consent requirement was waived by the Research Ethics Committee of Public Health (ref. 20220408/02) because the project was developed under the epidemiological surveillance competencies of the VC Ministry of Health (Law 10/2014 of the Valencian Community on Public Health).

### 2.2 Variables and definitions

Registered, prior SARS‐CoV‐2 infection was established by consulting the VC microbiology registry (RedMiVa) and taking into account the results of SARS‐CoV‐2 antibody assays performed, as described below. The vaccination status of participants was obtained from the VC vaccination registry. Participants were grouped into the following categories: vaccinated/experienced (VAC‐ex, vaccinated individuals with a record of a positive AIDT—active infection diagnostic test—result and/or serological evidence of past infection), vaccinated/naïve (VAC‐n, vaccinated participants with no record or serological evidence of previous SARS‐CoV‐2 infection), unvaccinated/experienced (UNVAC‐ex, unvaccinated population with history or serological evidence of prior SARS‐CoV‐2 infection) and unvaccinated/naïve (UNVAC‐n, unvaccinated population with no history or serological evidence of prior SARS‐CoV‐2 infection). AIDTs included rapid antigen‐detection assays targeting the N protein or commercially‐available RT‐PCRs.

### 2.3 Immunological testing

Serum SARS‐CoV‐2‐RBD‐total antibodies and nucleocapsid (N)‐reactive IgG antibodies were measured using the Roche Elecsys® Anti‐SARS‐CoV‐2 S and Elecsys® Anti‐SARS‐CoV‐2 N assays (Roche Diagnostics), respectively. Neutralizing antibodies (NtAb) targeting the S protein were measured using a GFP‐expressing vesicular stomatitis virus pseudotyped with the Wuhan‐Hu‐1 and Omicron BA.1 and BA.2 variants, as previously described^10^ (Supplementary Material). SARS‐CoV‐2‐S specific‐IFNγ‐producing CD4^+^ and CD8^+^ T cells were enumerated by whole‐blood flow cytometry for intracellular cytokine staining ICS (BD Fastimmune, Becton Dickinson and Company Biosciences, San Jose, CA), as previously reported^10^, ^11^ (Supplementary Material).

### 2.4 Statistical analysis

Frequency comparisons for categorical variables were carried out using the Fisher exact test. Differences between medians were compared via the Mann–Whitney U, Wilcoxon or Kruskal–Wallis test, as appropriate. Two‐sided exact *p*‐values were reported; a *p*‐value < 0.05 was considered statistically significant. The overall results are shown for the sample and weighted by the proportion of VC population in each age/sex stratum. The analyses were performed using SPSS version 20.0 (SPSS) and STATA 17.0 (StataCorp).

## 3 RESULTS

### 3.1 Participants' characteristics

Of 1043 eligible participants, 935 (89.6%) subjects—524 (56%) females and 411 (44%) males—aged between 0 and 87 years were finally enrolled in the study (Supporting Information: Table S1). The nonattendance of participants matching predetermined requirements regarding sex and age prevented full recruitment. The number of participants from each VC province was balanced to the respective population size. Detailed information regarding the vaccine platforms used and the number of doses received by participants is shown in Supporting Information: Table S2. In summary, 812 (86.8%) participants had received a complete vaccination schedule (in most cases, Comirnaty® or Spikevax®), 39 (4.1%) were incompletely vaccinated and 84 (8.9%) were unvaccinated (48% children aged under 9 years) at the time of recruitment. The percentage of fully vaccinated participants increased with age. Booster vaccine doses were administered to 582 (69%) participants (homologous in 276 and heterologous in 306) (Table 1). The median time elapsed between last vaccine dose and recruitment was 118 days (range: 2–428).

**Table 1:** COVID‐19 Vaccination status among participants according to their age

Age groups (no. of participants)	Vaccination status					
	No. of participants (%) that received an incomplete vaccine schedule	No. of participants (%) that received a complete vaccine schedule	No. of participants that received booster COVID‐19 vaccine dose/s (%)			Nonvaccinated participants (%)
			All combined	Heterologous	Homologous	
All ages (935)	39 (4)	812 (89)	582 (69)	306 (52□5)	276 (47□5)	84 (9)
0–9 (40)	13 (33)	8 (20)	0	‐	‐	19 (48)
10–19 (88)	10 (11)	68 (77)	9 (12)	7 (78)	2 (22)	10 (11)
20–34 (150)	10 (7)	126 (84)	63 (46)	50 (79)	13 (21)	14 (9)
35–49 (181)	4 (2)	158 (87)	103 (64)	79 (77)	24 (23)	19 (10)
50–64 (174)	1 (1)	169 (97)	135 (79)	114 (84)	21 (16)	4 (2)
65–79 (160)	1 (1)	152 (95)	145 (95)	48 (33)	97 (67)	7 (4)
>80 (142)	0 (0)	131 (92)	127 (97)	8 (6)	119 (94)	11 (8)

### 3.2 Cumulative incidence of SARS‐CoV‐2 infection

Blood volume was insufficient for analysis in four participants, who were accordingly excluded, leaving 931 individuals for the analyses detailed in continuation. Past SARS‐CoV‐2 infection was documented in a total of 442 (47.4%) participants (Table 2 and Supporting Information: Table S3), based on the presence of anti‐SARS‐CoV‐2‐N IgGs (*n* = 404), a recorded positive AIDT result (*n* = 237) or isolated detection of anti‐SARS‐CoV‐2‐RBD total antibodies in unvaccinated/SARS‐CoV‐2‐naïve individuals (*n* = 16). The crude cumulative incidence of prior SARS‐CoV‐2 infection in the VC was 47.4% (95% confidence interval [CI]: 44.1–50.6), whereas age/sex weighted cumulative incidence was 51.9% (95% CI: 48.7–55.1). The rate of past SARS‐CoV‐2 infection was inversely related to age; evidence of prior SARS‐CoV‐2 infection was found in more than two‐thirds of subjects under 34 years of age, in contrast to around a quarter of those over 65. Importantly, among participants with a documented positive AIDT result (*n* = 237), 11% were diagnosed with SARS‐CoV‐2 infection in 2020 (*n* = 25), 22% in the first semester of 2021 (*n* = 52), 17% in the second semester of 2021 (*n* = 40) and 51% in the first trimester of 2022 (*n* = 120), periods when the ancestral Wuhan‐Hu‐1 (including G614), Alpha, Delta and Omicron BA.1 variants, respectively, were dominant in the VC. Overall, the median time elapsed between microbiological diagnosis of SARS‐CoV‐2 infection and recruitment was 111 days (range: 8–591). For analytical purposes, participants were grouped as VAC‐ex (*n* = 379), VAC‐n (*n* = 472), UNVAC‐ex (*n* = 63), and UNVAC‐n (*n* = 21).

**Table 2:** Cumulative incidence of past SARS‐CoV‐2 infection, as determined by active infection diagnostic test and/or serological assays by participants' age

Age (years) group (no. of participants)	Record of a positive AIDT result (%; 95% CI)	Detectable anti‐SARS‐CoV‐2 N IgG (%; 95% CI)	Detectable anti‐SARS‐CoV‐2 RBD total antibodies (%; 95% CI)	Past SARS‐CoV‐2 infection (%; 95% CI)^a^
0–9 (40)	35.0 (20.6–51.7)	62.4 (46.7–78.1)	80.0 (67.0–93.0)	65.0 (48.3–79.4)
10–19 (87)	29.9 (20.5–40.6)	65.6 (55.4–75.8)	98.8 (96.6–100)	69.0 (58.1–78.4)
20–34 (150)	39.3 (31.5–47.6)	61.2 (53.3–69.1)	97.3 (94.7–99.9)	64.0 (55.8–71.7)
35–49 (181)	32.0 (25.3–39.4)	51.2 (43.8–58.5)	98.3 (96.5–100)	56.9 (49.3–64.2)
50–64 (173)	27.2 (20.7–34.4)	44.5 (37.0–52.0)	98.3 (96.3–100)	47.4 (39.8–55.1)
65–79 (159)	11.9 (7.3–18.0)	22.0 (15.5–28.6)	98.7 (97.0–100)	26.4 (19.7–34.0)
≥80 (141)	9.2 (5.0–15.2)	18.4 (11.9–24.8)	97.2 (94.3–99.9)	22.7 (16.1–30.5)
Total (931)	25.3 (22.6–28.3)	43.4 (40.2–46.6)	97.3 (96.3–98.4)	47.4 (44.1–50.6)
Total‐_W_ (931)^b^	28.2 (25.3–31.1)	47.9 (44.7–51.1)	96.7 (95.5–97.8)	51.9 (48.7–55.1)

Abbreviations: AIDT, active infection diagnostic test (antigen or RT‐PCR‐bases assays); CI, confidence interval; N, SARS‐CoV‐2 nucleocapsid protein; RBD, receptor binding domain.

^a^One or more of the following: positive AIDT result, detectable anti‐SARS‐CoV‐2 N IgG or detectable anti‐SARS‐CoV‐2 RBG total antibodies in unvaccinated participants (in the absence of anti‐N IgG, and history of a positive AIDT test.

^b^Total age/sex weighted Valencia Community population.

### 3.3 Anti‐SARS‐CoV‐2‐RBD total antibodies

**Figure 1:**
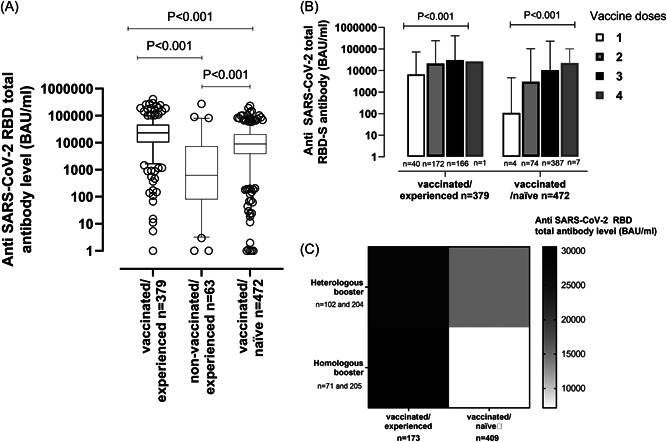
SARS‐CoV‐2‐receptor binding domain (RBD) total antibodies (in BAU/ml) in participants. (A) Box Whisker plots depicting serum antibody levels according to vaccination and SARS‐CoV‐2 infection status at time of recruitment. (B) Box Whisker plots depicting serum antibody levels in vaccinated/SARS‐CoV‐2‐experienced and vaccinated/SARS‐CoV‐2‐naïve participants by number of vaccine doses received. (C) Serum antibody levels in vaccinated/SARS‐CoV‐2‐experienced and vaccinated/SARS‐CoV‐2‐naïve receiving one or more booster doses by booster type (homologous vs. heterologous). *p*‐values for statistical comparisons are shown where appropriate.

Overall, 906/931 (97.3%) had detectable anti‐RBD total antibodies; most individuals (23/25) with undetectable antibody levels were UNVAC‐n children. As shown in Figure 1A, median anti‐RBD total antibody levels were significantly higher (*p* < 0.001) in VAC‐ex than in VAC‐n participants (median: 23,120 BAU/ml; IQR: 10,186–46,196 vs. median: 9,023 BAU/ml; IQR: 3844–20,329); this difference was observed across most age ranges (Supporting Information: Figure S1). In turn, VAC‐ex and VAC‐n individuals displayed significantly higher (*p* < 0.001) antibody levels than UNVAC‐exparticipants (median: 622 BAU/ml; IQR: 82–6913).

Compared with VAC‐n, individuals in the VAC‐ex subgroup were younger (median: 41 years; range: 5–94 years; vs. 62 years; range: 5–95; *p* < 0.001) and more frequently men (49% vs. 40%; *p* < 0.02). Importantly, time elapsed between last vaccine dose and testing was shorter in VAC‐n than in VAC‐ex subjects (median: 116 days; range: 0–428 vs. 150 days; range: 0–343; *p* < 0.001), as the former were more likely (*p* < 0.001) to have received a booster vaccine dose than the latter (83.5% vs. 44.3%). In turn, VAC‐ex and UNVAC‐ex participants were matched for sex (*p* = 0.62) and time since diagnosis of SARS‐CoV‐2 infection (median: 92 days; range: 8–718; vs. median: 82 days; range: 51–516; *p* = 0.25). Nevertheless, the UNVAC‐ex subgroup comprised more participants aged under 9 years than the VAC‐ex group, which included more subjects aged 50–64 years (*p* < 0.001).

Overall, the number of vaccine doses received had a direct impact on the level of anti‐RBD total antibodies, irrespective of SARS‐CoV‐2 infection status (Figure 1B). Interestingly, among participants who had received a third vaccine dose, heterologous booster shots resulted in increased anti‐RBD antibody levels compared with homologous schedules in SARS‐CoV‐2‐naïve, but not SARS‐CoV‐2‐experienced participants, regardless of age and sex (Figure 1C).

**Figure 2:**
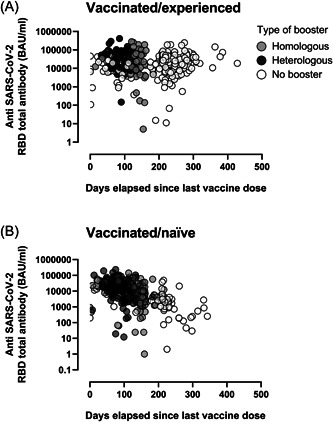
SARS‐CoV‐2‐receptor binding domain (RBD) total antibodies (in BAU/ml) in vaccinated/SARS‐CoV‐2‐experienced (A) and vaccinated/SARS‐CoV‐2‐naïve (B) participants according to the vaccination schedule (full vaccination with no booster dose, homologous booster dose and heterologous booster dose) and time elapsed since last vaccine dose.

**Figure 3:**
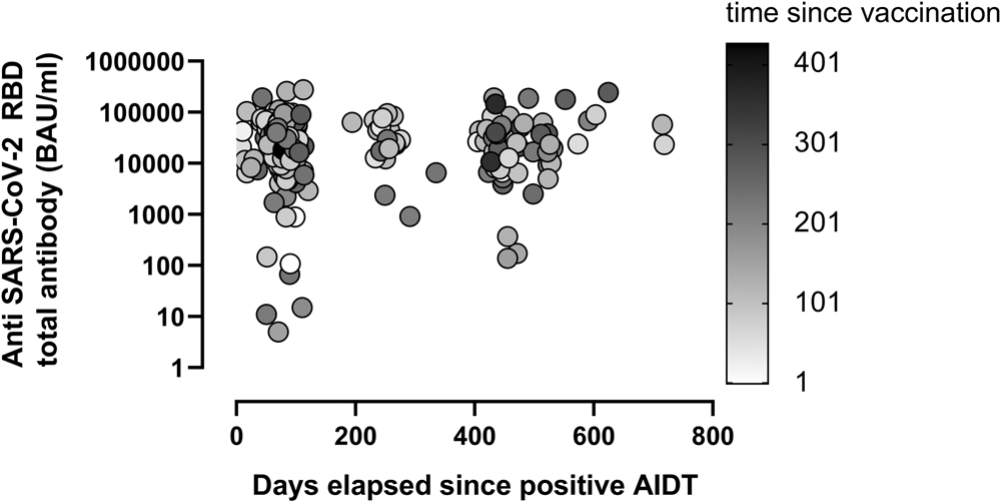
SARS‐CoV‐2‐receptor binding domain (RBD) total antibodies (in BAU/ml) in vaccinated/SARS‐CoV‐2 experienced participants with a history of a positive active infection diagnostic test (AIDT) according to time elapsed since documentation of the positive result and time since last vaccine dose.

However, no correlation was found between time elapsed since last vaccine dose and anti‐RBD total antibody levels (rho: –0.02; 95% CI: –0.13 to 0.07; *p* = 0.57) in VAC‐ex individuals, whereas a moderate inverse correlation (Rho: –0.52; 95% CI: –0.59 to –0.45; *p* < 0.001) was observed in VAC‐n (Figure 2). Interestingly, in VAC‐ex participants with a recorded positive AIDT result (*n* = 220) (Figure 3), time elapsed since documentation of SARS‐CoV‐2 infection correlated weakly (inversely) with anti‐RBD antibody levels (Rho: –0.30; 95% CI: –0.42 to –0.17; *p* < 0.001).

Finally, subtle age‐related differences in anti‐RBD total antibody levels were noticed in both VAC‐ex and VAC‐n subgroups (Supporting Information: Figure S1), which were likely related to dissimilarities across age groups regarding the number of vaccine shots received and time elapsed since administration of the last vaccine dose.

### 3.4 Neutralizing antibodies against the ancestral Wuhan‐Hu‐1 and Omicron subvariants

A total of 100 randomly selected participants across all age groups were screened for the presence of NtAb against SARS‐CoV‐2 (sub)variants. The main characteristics of the subjects evaluated are shown in Supporting Information: Table S4.

The proportions of participants presenting NtAbs against the Wuhan‐Hu‐1 variant were 100%, 93%, and 40% in the VAC‐ex, VAC‐n and UNVAC‐ex groups, respectively; for Omicron BA.1, the percentages were 94%, 75%, and 50%, respectively; and for Omicron BA.2, 97%, 84%, and 40%, respectively.

**Figure 4:**
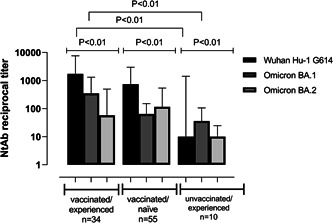
Serum neutralizing antibody titers (reciprocal IC_50_) against SARS‐CoV‐2 Wuhan‐Hu‐1, Omicron BA.1 and BA.2 (sub)variants in vaccinated/SARS‐CoV‐2 experienced, vaccinated/SARS‐CoV‐2 naïve participants and unvaccinated/SARS‐CoV‐2 experienced participants. Vaccinated/experienced, vaccinated/naïve and nonvaccinated/experienced participants were matched for sex and age, and vaccinated participants for booster type. Nevertheless, time since last vaccine dose was significantly shorter (*p* < 0.001) in vaccinated/naïve than in vaccinated/experienced participants (median 116 days; range, 29–298; vs. 162 days; range, 0–308). *p*‐values for statistical comparisons are shown.

Overall, NtAb titers were lower against Omicron BA.1 and BA.2 than against Wuhan‐Hu‐1 across all comparison groups; in turn, median levels of NtAb binding Omicron BA.1 and BA.2 were comparable across all study groups (Figure 4). Of note, NtAb targeting all (sub)variants screened were higher in VAC‐ex than in VAC‐n individuals, although statistical significance was reached for those against Wuhan‐Hu‐1 (*p* = 0.05) and BA.1 (*p* = 0.008), but not against BA.2 (*p* = 0.74) (Figure 4). Moreover, both VAC‐ex and VAC‐n displayed higher NtAb titers than UNVAC‐ex participants (*p* = 0.001 for Wuhan Hu‐1; *p* = 0.052 for BA.1 and *p* = 0.003 for BA.2). Overall, comparable NtAb titers against all (sub)variants tested were seen across VAC‐ex participants whether boosted with an additional vaccine dose or not (Supporting Information: Table S5). Nevertheless, a significant, although weak, inverse correlation between NtAb titers against Wuhan‐Hu‐1 and Omicron BA.1 and time elapsed since last vaccine dose was observed in VAC‐n but not VAC‐ex individuals (Supporting Information: Table S6).

### 3.5 SARS‐CoV‐2‐Spike T‐cell responses

**Figure 5:**
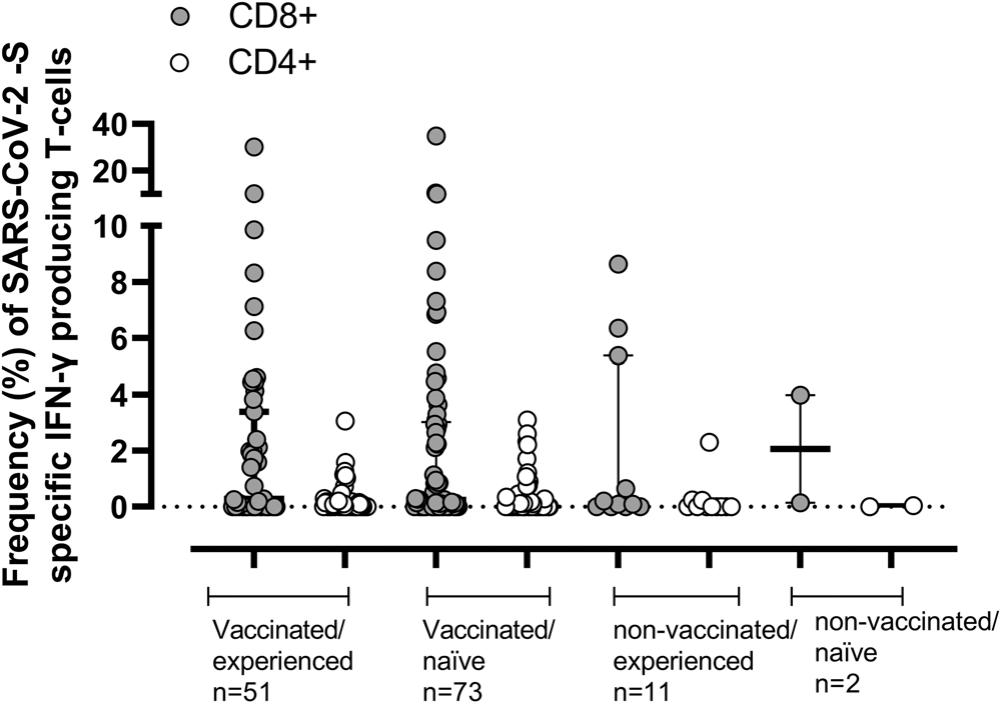
Frequency of SARS‐CoV‐2‐Spike(S)‐reactive IFN‐ γ‐producing CD4^+^ and CD8^+^ T cells in vaccinated/SARS‐CoV‐2 experienced, vaccinated/SARS‐CoV‐2 naïve participants, unvaccinated/SARS‐CoV‐2 experienced and unvaccinated/SARS‐CoV‐2 naïve participants. Vaccinated/experienced, vaccinated/naïve and unvaccinated/experienced participants were balanced for sex and age, and for vaccinated individuals for booster type; nevertheless, time since last vaccine dose was significantly lower (*p* < 0.001) in vaccinated/naïve than in vaccinated/experienced participants (median 113 days; range, 0–298; vs. 175 days; range, 0–308).

We enumerated SARS‐CoV‐2‐S‐reactive IFN‐γ CD4^+^ and CD8^+^ T cells in 137 participants (Supporting Information: Table S4). Overall, 101 (73.7%) participants had detectable SARS‐CoV‐2‐S‐IFN‐ γ T cells: CD4^+^ (*n* = 15; 14.9%), CD8^+^ (*n* = 41; 40.6%) or both (*n* = 45; 44.6%). In detail, the figures were 73%, 75%, and 64% for VAC‐ex, VAC‐n, and UNVAC‐ex, respectively. Median frequencies of both T‐cell subsets were comparable across subgroups (*p* = 0.92 for CD8^+^ T cells and *p* = 0.80 for CD4^+^ T cells) (Figure 5). Likewise, neither age (*p* = 0.86) nor sex (*p* = 0.57) impacted the median frequencies of the two T‐cell subsets. SARS‐CoV‐2 T‐cell subset frequencies were comparable irrespective of having received booster vaccine doses (Supporting Information: Table S5), or time elapsed since last vaccine dose (Supporting Information: Table S6) in both VAC‐ex and VAC‐n individuals.

## 4 DISCUSSION

To date, only a handful of seroepidemiological studies extending into the Omicron wave have been performed in the general population.^4^, ^5^, ^6^ Here, we conducted a cross‐sectional serosurvey to estimate the prevalence of SARS‐CoV‐2 antibodies in the general VC population after the Omicron BA.1 variant surge and retrieved prior documented SARS‐CoV‐2 infection data for the recruited population from the RedMiVa VC registry. Our results can probably be extrapolated to other autonomous communities in Spain, as previously suggested.^3^ We estimated an age/sex weighted cumulative incidence of prior SARS‐CoV‐2 infection of 51.9% (95% CI: 48.7–55.1). While this figure was comparable across males and females, age‐related differences were observed, the highest rate occurring in individuals under 34 years of age and the lowest in those aged 65 or older. Such differences could be explained by dissimilarities in the rate of full or booster vaccination and social behavior across age groups. Interestingly, around 9% of participants lacked anti‐SARS‐CoV‐2‐N antibodies despite documentation of previous SARS‐CoV‐2 infection, suggesting, in line with previous findings,^12^ that assessment of anti‐N antibodies alone might underestimate the incidence of SARS‐CoV‐2 infection.

Evaluation of SARS‐CoV‐2 immune status according to vaccination schedule, time elapsed since vaccination, and previous virus exposure also provides valuable information to model the course of the pandemic. Here, we measured serum anti‐RBD total antibody levels, as a surrogate for serum neutralizing activity,^6^, ^7^ in all participants, and NtAb titers and the frequency of peripheral blood SARS‐CoV‐2‐S‐reactive IFN‐ γ CD4^+^ and CD8^+^ T cells in a randomly selected subset representing the entire study population. In our view, the following facts were relevant for data interpretation. First, the median time elapsed from last vaccine dose to testing was around 4 months; second, among participants with a documented positive AIDT result, the median time elapsed between SARS‐CoV‐2 diagnosis and recruitment was 111 days overall; third, as stated above, approximately 50% of SARS‐CoV‐2 infections in recruited subjects with a positive AIDT result were seemingly caused by the Omicron BA.1 variant.

In this setting, 97% of participants had detectable anti‐RBD total antibodies, whose levels were increased in VAC‐ex individuals compared with VAC‐n participants and, especially, with UNVAC‐ex individuals matched for time of SARS‐CoV‐2 diagnosis, suggesting that antibody waning may occur more rapidly in these latter individuals. VAC‐ex participants displayed higher levels of anti‐RBD total antibodies than Vac‐n across virtually all age groups. This occurred irrespective of the number of vaccine doses received and despite VAC‐n having received the last vaccine dose (in most cases a booster) more recently than VAC‐ex subjects. Importantly, the increase in anti‐RBD total antibodies following a booster vaccine dose was greater in VAC‐n than VAC‐ex participants, particularly after a heterologous boost, in line with previous findings.^13^, ^14^ Our data suggested that anti‐RBD total antibody responses may be more durable in VAC‐ex, as evidenced by an inverse correlation between time elapsed since last vaccine dose and anti‐RBD total antibody levels in VAC‐n but not VAC‐ex participants. In addition, time elapsed since documentation of SARS‐CoV‐2 infection correlated weakly (inversely) with anti‐RBD antibody levels in VAC‐ex participants. Taken collectively, this data suggested that hybrid immunity provided stronger and more durable anti‐RBD antibody responses than vaccination alone, which concurs with previous observations.^12^, ^15^, ^16^, ^17^, ^18^, ^19^, ^20^, ^21^

Our data on virus neutralization experiments led to several potentially relevant conclusions. First, the proportion of participants displaying detectable NtAbs against Omicron BA.2, the dominant variant in the VC by May 2022, before completion of the current study, was as high as that against Wuhan‐Hu‐1 and Omicron BA.1; moreover, median levels of NtAbs binding Omicron BA.1 and BA.2 were comparable in participants across all study subgroups, suggesting a substantial degree of B‐cell epitope cross‐reactivity across these subvariants, as previously observed.^22^ Our finding that a high percentage of vaccinated participants had either prior SARS‐CoV‐2 infection or detectable NtAbs against Omicron BA.1 (94% and 75%, respectively) was compatible with the idea that both Wuhan‐based vaccine platforms and breakthrough infections elicit cross‐reactive antibodies against SARS‐CoV‐2 variants of concern^14^, ^23^ and that Omicron BA.1 breakthrough infection may induce sublineage‐specific (i.e., BA.2) NtAbs, in addition to recall of memory B cells established by prior vaccination.^24^, ^25^, ^26^ Second, our data supported the assumption that hybrid immunity provides a more robust and, perhaps, more durable NtAb response against Wuhan‐Hu‐1 and Omicron BA.1 than that elicited by either vaccination or natural infection alone. Nevertheless, this was apparently not the case for BA.2; this could have important implications in the design of vaccination policies in the near future, as currently dominant sublineages worldwide, such as BA.4, BA.5, BA.2.12.1, and BA.2.75 resemble BA.2 more than BA.1 in terms of their ability to escape antibody responses elicited by Wuhan‐Hu‐1 based vaccines.^13^ Third, our data revealed no differences in NtAb titers against all (sub)variants tested across VAC‐ex participants, boosted or not with an additional vaccine dose. Nevertheless, the low number of participants included in the analysis limited the robustness of this conclusion. Detectable SARS‐CoV‐2 T‐cell responses are known to persist up to 8 months following natural infection or vaccination.^27^, ^28^, ^29^ Our data supported this notion in that SARS‐CoV‐2‐S‐ IFN‐γ CD4^+^, CD8^+^ T cells, or both could be detected in 75% of participants a median of 4 months after the last vaccine dose. In effect, SARS‐CoV‐2‐S IFN‐γ T‐cell responses may remain relatively stable over time as frequencies of both T‐cell subsets did not correlate with time elapsed since last vaccine dose. Moreover, administering a booster dose had minimal impact on the magnitude of T‐cell responses. Interestingly, in contrast to our observations on functional antibodies, we found no differences between VAC‐ex and VAC‐n regarding the frequencies of either T‐cell subset.

A limitation of our study is that it lags behind the emerging SARS‐CoV‐2 subvariant epidemiological landscape in the VC and worldwide; yet the data on the cumulative incidence of SARS‐CoV‐2 infection across the different age groups revealed a virus transmission pattern in our community, which has probably been maintained following the BA.5 and BA.2.12.1 surges (in Spain, the presence of BA.2.75 is currently anecdotical). This may allow public health officials and policymakers to identify hot spots and areas where transmission has been reduced so that they may respond accordingly (selective administration of vaccine boosters or targeted use of monoclonal antibodies, when indicated). Also, our data could help establish selective mitigation tactics upon the emergence and spread of new, highly transmissible sublineages. Our study had other limitations: first, bias may have been introduced by excluding some PCZs and by only including individuals scheduled for blood tests; second, participants were not asked if they had recently experienced COVID‐19‐related symptoms; Third, SARS‐CoV‐2 variant sequencing was not performed; Fourth, an unstandardized flow cytometry ICS assay was used for enumerating SARS‐CoV‐2‐S‐reactive T cells; Fifth, T‐cell functionalities other than IFN‐γ production were not explored and Wuhan‐Hu‐1‐based instead of VOC‐adapted overlapping peptide libraries were used.

In summary, our study indicated that, by April 2022, around half of the VC population had been infected with SARS‐CoV‐2 and, due to extensive vaccination, exhibited hybrid immunity. Currently, the BA.5 omicron sublineage dominates in the VC. The large percentage of participants with detectable functional antibody and T‐cell responses against SARS‐CoV‐2, which may be cross‐reactive with this subvariant to some extent, indicated lower expected severity than in previous waves. Although pending confirmation in wider studies, this aspect is relevant for public health decision‐making.

## AUTHOR CONTRIBUTIONS

Jorge Camacho, Estela Giménez, Eliseo Albert, Joao Zulaica, Ignacio Torres, Luciana Rusu, Beatriz Álvarez Rodríguez, María Jesús Alcaraz, Iñaki Comas, Fernando Gonzáles‐Candelas, and Ron Geller carried out antibody and T‐cell assays and validated and analyzed the data. Ramón Limón was responsible for the logistics of the study. Javier S. Burgos, Hermelinda Vanaclocha, Salvador Peiró, José Sánchez‐Payá, Javier Díez‐Domingo, and David Navarro: conceptualization, study design, and data interpretation. Salvador Peiró and David Navarro wrote the manuscript. All authors reviewed the final version of the manuscript.

## CONFLICT OF INTEREST

The authors declare no conflict of interest.

## Supporting information

Supplementary Figure 1. SARS‐CoV‐2‐receptor binding domain (RBD) total antibodies (in BAU/ml) in vaccinated/SARS‐CoV‐2 experienced (A) and vaccinated/SARS‐CoV‐2 naïve (B) participants, according to age. *p*‐values for statistical comparisons are shown.

## Data Availability

The data that support the findings of this study are available from the corresponding author upon reasonable request.
